# ﻿The diversity and taxonomy of *Tomentella* (Thelephoraceae, Thelephorales) with descriptions of four new species from Southwestern China

**DOI:** 10.3897/mycokeys.109.132941

**Published:** 2024-09-17

**Authors:** Xiaojie Zhang, Fulei Shi, Ke Yang, Changlin Zhao

**Affiliations:** 1 The Key Laboratory of Forest Resources Conservation and Utilization in the Southwest Mountains of China Ministry of Education, Key Laboratory of National Forestry and Grassland Administration on Biodiversity Conservation in Southwest China, Yunnan Provincial Key Laboratory for Conservation and Utilization of In-forest Resource, Southwest Forestry University, Kunming 650224, China; 2 College of Forestry, Southwest Forestry University, Kunming 650224, China; 3 Management and Conservation Bureau, Wumeng Mountain National Nature Reserve, Zhaotong 657000, China

**Keywords:** Biodiversity, China, phylogenetic analyses, taxonomy, wood-inhabiting fungi, Yunnan Province

## Abstract

Taxonomy plays a central role in understanding the diversity of life, translating the products of biological exploration and discovery specimens and observations into systems of names that settle a “classification home” to taxa. Up to this point, studies on the taxonomy and phylogeny of the basidiomycetous genus *Tomentella* stemmed mainly from the temperate to boreal zones of the North Hemisphere, but were scarce in tropical Asia. In this study, four new species, *viz. Tomentellaolivaceobasidiosa*, *T.velutina*, *T.wumenshanensis* and *T.yunnanensis* from China, are described and illustrated based on the morphological characteristics and molecular phylogenetic analyses, in which the sequences of ITS+nLSU+mtSSU+RPB2 genes were used for the phylogenetic analyses using Maximum Likelihood, Maximum Parsimony and Bayesian Inference methods. All the new species can be well recognised by their macroscopical and anatomical characteristics. The four new species, closely related taxa in the phylogenetic tree and morphologically similar species are discussed.

## ﻿Introduction

The genera *Amaurodon* J. Schröt., *Odontia* Pers., *Pseudotomentella* Svrcek, *Tomentella* Pers. ex. Pat. and *Tomentellopsis* Hjortstam, belong to the family Thelephoraceae Chevall. of the order Thelephorales Corner ex Oberw. and the phylum Basidiomycota R.T. Moore (Reid and Larsen 1976; Kõljalg 1996). As their common morphological characteristics are resupinate and thin basidiomata, they were recognised as the resupinate thelephoroid fungi by Kõljalg (1996). Species of the group have their own typical characteristics, such as the light blue basidiomata of *Amaurodon*, the granulose or hydnoid hymenial surface of *Odontia*, the basidiospores with bifurcate warts or spines of *Pseudotomentella* and the absence of rhizomorphs in the genus *Tomentellopsis*. However, the genus *Tomentella* has diverse and complex morphological features, such as basidiomata with various colours and smooth to granulose surfaces, and basidiospores with diverse shapes and ornamentations (Reid and Larsen 1976; Kõljalg 1996).

*Tomentella* species have been recognized as ectomycorrhizal (ECM) fungi since the 1980s (Danielson et al. 1983; Kõljalg et al. 2001; Tedersoo et al. 2014). In various of forest ecosystems, the ECM*Tomentella*-*Thelephora* lineages are amongst the richest species (Tedersoo et al. 2014; Jakucs et al. 2015; Nouhra et al. 2015). As ectomycorrhizal fungi, they play an important role in nutrient cycling and ecological functions in forest ecosystems (Read and Perez-Moreno 2003; Jakucs et al. 2015; Nouhra et al. 2015). *Tomentella* species formed ectomycorrhiza with many host tree families, including Achatocarpaceae, Apocynaceae, Betulaceae, Cistaceae, Dipterocarpaceae, Ericaceae, Fabaceae, Fabaceae subfamily Caesalpinioideae, Fagaceae, Orchidaceae, Pinaceae, Myrtaceae, Nothofagaceae, Nyctaginaceae, Papilionoideae, Polygonaceae, Pyrolaceae, Rhamnaceae, Rosaceae, Salicaceae, Ticodendraceae and Tiliaceae (Tedersoo et al. 2007, 2008; Smith et al. 2011; Jakucs et al. 2015; Salomón et al. 2017; Alvarez-Manjarrez et al. 2018; Malysheva et al. 2018; Põlme 2018). Ranging from temperate to tropical zones in forests, the basidiomata of *Tomentella* are often found on fallen branches and leaves with decayed coniferous and deciduous wood debris, bark, soil, twigs, stumps, stone or even charred wood (Reid and Larsen 1976; Kõljalg 1996; Kuhar et al. 2016).

The genus *Tomentella* was sister to *Thelephora* in which both are nested within the family Thelephoraceae, while the morphological limits between *Tomentella* and *Thelephora* are not yet clear (Patouillard 1887; Ezhov and Zmitrovich 2017). In previous scientific research, studies about wood-inhabiting fungal molecular systematics revealed that within the species with different macroscopic characteristics located in the same family or even within the same genus, similar microscopic characteristics can be seen (Cui et al. 2019; Guan and Zhao 2021; Guan et al. 2021; Wu et al. 2022; Guan et al. 2023; Wang et al. 2023; Zhao et al. 2023b, 2024; Luo et al. 2024). The studies showed that ITS or nLSU sequences alone could not resolve the phylogenetic relationships in this complex group (*Thelephora*/*Tomentella* clade) (Patouillard 1887; Vizzini et al. 2016; Ezhov and Zmitrovich 2017). The research mentioned that genera *Thelephora* and *Tomentella* will be considered one genus, based on the morphological and phylogenetic results.

This work describes four new species of *Tomentella*, which were found in southwest China, based on the morphology and phylogeny. It provides full descriptions, colour photographs, a detailed comparison of four new species with closely related taxa and phylogenetic trees showing the placement of four new species within the genus *Tomentella*.

## ﻿Materials and methods

### ﻿Morphology

Fresh fruiting bodies of the fungi were collected from Kunming, Lincang and Zhaotong of Yunnan Province, P.R. China. Specimens were dried in an electric food dehydrator at 40 °C (Hu et al. 2022), then sealed and stored in an envelope bag and deposited in the herbarium of the Southwest Forestry University (SWFC), Kunming, Yunnan Province, P.R. China. Macromorphological descriptions are based on field notes and photos captured in the field and lab. Colour terminology follows Petersen (Petersen 1996). Micromorphological data were obtained from the dried specimens when observed under a light microscope following the previous study (Guan et al. 2023; Zhao et al. 2023a). The following abbreviations are used: **KOH** = 5% potassium hydroxide water solution, **CB** = Cotton Blue, **CB–** = acyanophilous, **IKI** = Melzer’s Reagent, **IKI–** = both inamyloid and indextrinoid, **L** = mean spore length (arithmetic average for all spores), **W** = mean spore width (arithmetic average for all spores), **Q** = variation in the L/W ratios between the specimens studied and **n** = a/b (number of spores (a) measured from given number (b) of specimens).

### ﻿Molecular phylogeny

The EZNA HP Fungal DNA Kit (Omega Biotechnologies Co., Ltd., Kunming, China) was used to extract DNA with some modifications from the dried specimens. The nuclear ribosomal ITS region was amplified with primers ITS5 and ITS4 (White et al. 1990). The PCR procedure for ITS was as follows: initial denaturation at 95 °C for 3 min, followed by 35 cycles at 94 °C for 40 s, 58 °C for 45 s and 72 °C for 1 min and a final extension of 72 °C for 10 min. The nuclear nLSU region was amplified with primer pair LR0R and LR7 (Vilgalys and Hester 1990; Rehner and Samuels 1994). The PCR procedure for nLSU was as follows: initial denaturation at 94 °C for 1 min, followed by 35 cycles at 94 °C for 30 s, 48 °C for 1 min and 72 °C for 1.5 min and a final extension of 72 °C for 10 min. The nuclear mt-SSU region was amplified with primer pair MS1 and MS2 (White et al. 1990). The PCR procedure for mt-SSU was as follows: initial denaturation at 94 °C for 2 min, followed by 36 cycles at 94 °C for 45 s, 52 °C for 45 s and 72 °C for 1 min and a final extension of 72 °C for 10 min. The nuclear RPB2 region was amplified with primer pair bRPB2-6F and bRPB2-7.1R (Matheny 2005). The PCR procedure for RPB2 was as follows: initial denaturation at 95 °C for 2.5 min, denaturation at 95 °C for 30 s, annealing at 52 °C for 1 min, extension at 72 °C for 1 min (add 1 °C per cycle), repeat for 40 cycles starting at step 2, extension at 72 °C for 1.5 min, repeat for 40 cycles starting at step 6, leave at 72 °C for 5 min. The PCR products were purified and directly sequenced at Kunming Tsingke Biological Technology Limited Company, Yunnan Province, China. All newly generated sequences were deposited in NCBI GenBank (https://www.ncbi.nlm.nih.gov/genbank/) (Table 1).

**Table 1.** List of species, specimens and GenBank accession numbers of sequences used in this study. New species is shown in bold.

Species name	Specimen No.	GenBank accession No.				Country	References
		ITS	nLSU	mt-SSU	RPB2		
*Amaurodonaquicoeruleus*	UK452	AM490944	—	—	—	Australia	Miettinen and Kõljalg (2007)
*A.caeruleocaseus*	PERTH06670709	MT565478	—	—	—	Australia	Unpublished
*A.hydnoides*	TU108407	AM490941	—	—	—	Venezuela	Miettinen and Kõljalg (2007)
*Lenzitopsisdaii*	Yuan2952	JN169798	MT319136	—	—	China	Zhou and Kõljalg (2013)
*Odontiasparsa*	Yuan 10718	MG719980	—	—	—	China	Yuan et al. (2018)
*O.sparsa*	Yuan 10780	MG719979	—	—	—	China	Yuan et al. (2018)
*Phellinotusneoaridus*	URM 83203	MZ954858	MZ964977	—	—	Brazil	Salvador-Montoya et al. (2022)
*Phellodonatroardesiacus*	Cui 18449	MZ221189	MZ225598	MZ225636	—	China	Song et al. (2023)
*P.atroardesiacus*	Cui 18457	MZ225577	MZ225599	—	—	China	Song et al. (2023)
*P.cinereofuscus*	Cui 16962	MZ225583	MZ225605	MZ225643	MZ343200	China	Song et al. (2023)
*P.cinereofuscus*	Cui 16963	MZ225584	MZ225606	MZ225644	MZ343201	China	Song et al. (2023)
*P.melaleucus*	Cui 18614	OL449262	OL439032	OL439022	—	China	Song et al. (2023)
*P.melaleucus*	Cui 18620	OL449263	OL439033	OL439023	—	China	Song et al. (2023)
*P.yunnanensis*	Cui 17129	MZ225594	MZ225614	MZ225652	MZ343207	China	Song et al. (2023)
*P.yunnanensis*	Cui 17131	MZ225595	MZ225615	MZ225653	MZ343208	China	Song et al. (2023)
*Polyozellusatrolazulinus*	TU117559	MG214657	—	—	—	Canada	Voitk et al. (2018)
*P.atrolazulinus*	TU117477	MF100839	—	—	—	Canada	Voitk et al. (2018)
*P.mariae*	TU117235	MF100826	—	—	—	Canada	Voitk et al. (2018)
*P.purpureoniger*	TU103000	MF100821	—	—	—	USA	Voitk et al. (2018)
*Thelephoraganbajun*	Yuan16756	OP793761	OP793690	OP793718	—	China	Lu et al. (2022)
*T.ganbajun*	Yuan16817	OP793762	OP793687	OP793721	—	China	Lu et al. (2022)
*T.grandinioides*	CLZhao 3406	MZ400677	MZ400671	—	—	China	Liu et al. (2021)
*T.grandinioides*	CLZhao 3408	MZ400678	MZ400672	—	—	China	Liu et al. (2021)
*T.africana*	SYN 991	EF507254	—	—	—	Benin	Yorou and Agerer (2008)
*T.africana*	M SYN 991	NR_119637	—	—	—	Benin	Yorou and Agerer (2008)
*T.afrostuposa*	SYN 2292	JF520431	—	—	—	Guinea	Yorou et al. (2012b)
*T.afrostuposa*	M SYN 2292	NR_119954	—	—	—	Guinea	Unpublished
*T.agbassaensis*	M SYN 981	NR_119638	—	—	—	Benin	Unpublished
*T.agbassaensis*	SYN 981	EF507257	—	—	—	Benin	Yorou et al. (2012a)
*T.agereri*	RA 13793	EF538424	—	—	—	Benin	Yorou et al. (2011)
*T.agereri*	M RA 13793	NR_119641	—	—	—	Benin	Unpublished
*T.alpina*	IB 20060231	NR_121330	—	—	—	Australia	Unpublished
*T.amyloapiculata*	SYN 893	EF507263	—	—	—	Benin	Yorou et al. (2012a)
*T.amyloapiculata*	M SYN 893	NR_119639	—	—	—	Benin	Unpublished
*T.asperula*	iNat66942560	ON943290	—	—	—	Canada	Unpublished
*T.atrobadia*	Yuan 11099	—	MK446335	—	—	China	Yuan et al. (2020)
*T.atrobadia*	Yuan 11114	—	MK446336	—	—	China	Yuan et al. (2020)
*T.atrocastanea*	Yuan 12170	MK211742	MK446337	—	—	China	Yuan et al. (2020)
*T.atrocastanea*	Yuan 12179	MK211743	MK446338	—	—	China	Yuan et al. (2020)
*T.aureomarginata*	Yuan 10671	MK211744	MK446339	—	—	China	Yuan et al. (2020)
*T.aureomarginata*	Yuan 10683	MK211745	MK878395	—	—	China	Yuan et al. (2020)
*T.badia*	LE 299095	MT981507	—	—	—	Russia	Unpublished
*T.badia*	LE 314775	MT981499	—	—	—	Russia	Unpublished
*T.bidoupensis*	Yuan 12707	—	MN684329	—	—	Vietnam	Lu et al. (2022)
*T.bidoupensis*	Yuan 12685	—	MN684330	—	—	Vietnam	Lu et al. (2022)
*T.botryoides*	O-F256708	MT146455	—	—	—	Sweden	Svantesson et al. (2021)
*T.botryoides*	O-F256707	MT146454	—	—	—	Sweden	Svantesson et al. (2021)
*T.brevis*	Yuan 11328	—	MK446340	—	—	China	Yuan et al. (2020)
*T.brevis*	Yuan 11332	—	MK878396	—	—	China	Yuan et al. (2020)
*T.brevisterigmata*	IFP 019338	NR_185567	—	—	—	China	Unpublished
*T.brunneocystidia*	SYN 839	DQ848613	—	—	—	Benin	Yorou and Agerer (2007)
*T.brunneocystidia*	RA 13779	DQ848610	—	—	—	Benin	Yorou and Agerer (2007)
*T.brunneoflava*	Yuan 12162	MK211749	MK850194	—	—	China	Yuan et al. (2020)
*T.brunneoflava*	Yuan 12161	MK211748	—	—	—	China	Yuan et al. (2020)
*T.brunneogrisea*	Yuan 12147	—	MK446343	—	—	China	Yuan et al. (2020)
*T.bryophila*	FFP1020	JQ711917	—	—	—	Canada	Jones et al. (2012)
*T.capitata*	SYN 860	DQ848612	—	—	—	Benin	Yorou and Agerer (2007)
*T.capitata*	RA 13785	DQ848611	—	—	—	Benin	Yorou and Agerer (2007)
*T.capitatocystidiata*	Yuan 11459	—	MK446344	—	—	China	Yuan et al. (2020)
*T.capitatocystidiata*	Yuan 11494	—	MK446345	—	—	China	Yuan et al. (2020)
*T.casiae*	Yuan 18263	PP479638	PP486302	—	—	China	Zhu et al. (2024)
*T.casiae*	Yuan 18254	PP479637	PP486299	—	—	China	Zhu et al. (2024)
*T.castanea*	JW1	KC952674	—	—	—	Germany	Unpublished
*T.changbaiensis*	Yuan 11477	—	MK446346	—	—	China	Yuan et al. (2020)
*T.changbaiensis*	Yuan 11496	—	MK446347	—	—	China	Yuan et al. (2020)
*T.cinerascens*	SS301	MT146467	—	—	—	Sweden	Svantesson et al. (2021)
*T.cinerascens*	SP72a	OQ418570	—	—	—	Sweden	Svantesson et al. (2021)
*T.cinereobrunnea*	Yuan 12705	—	MK850199	—	—	Vietnam	Lu et al. (2022)
*T.cinereobrunnea*	Yuan 12703	—	MK850198	—	—	Vietnam	Lu et al. (2022)
*T.citrinocystidiata*	Yuan 10680	—	MK446348	—	—	China	Yuan et al. (2020)
*T.citrinocystidiata*	Yuan 10743	—	MK446349	—	—	China	Yuan et al. (2020)
*T.coerulea*	MFT22	MK431005	—	—	—	Germany	Unpublished
*T.coerulea*	MTB3	MN947340	—	—	—	Germany	Unpublished
*T.coffeae*	Yuan 10629	—	MK446350	—	—	China	Yuan et al. (2020)
*T.coffeae*	Yuan 11100	—	MK446351	—	—	China	Yuan et al. (2020)
*T.conclusa*	Yuan 11986	—	MK850195	—	—	China	Yuan et al. (2020)
*T.conclusa*	Yuan 12086	—	MK446352	—	—	China	Yuan et al. (2020)
*T.cystidiata*	Yuan 10620	—	MK446353	—	—	China	Yuan et al. (2020)
*T.cystidiata*	Yuan 10693	—	MK446354	—	—	China	Yuan et al. (2020)
*T.dimidiata*	Yuan 11205	MK211704	MK446355	—	—	China	Yuan et al. (2020)
*T.dimidiata*	Yuan 11267	MK211705	MK446356	—	—	China	Yuan et al. (2020)
*T.duplexa*	Yuan 12202	MK211706	MK446357	—	—	China	Yuan et al. (2020)
*T.duplexa*	Yuan 12207	MK211707	MK446358	—	—	China	Yuan et al. (2020)
*T.efibulata*	Yuan 11167	—	MK446360	—	—	China	Yuan et al. (2020)
*T.efibulata*	Yuan 10699	—	MK446359	—	—	China	Yuan et al. (2020)
*T.efibulis*	Yuan 11241	MK211708	MK446361	—	—	China	Yuan et al. (2020)
*T.efibulis*	Yuan 11329	MK211709	MK446362	—	—	China	Yuan et al. (2020)
*T.ellisii*	src846	DQ974775	—	—	—	USA	Smith et al. (2007)
*T.ferruginea*	LE F-332319	MT981501	—	—	—	Russia	Ivanushenko and Volobuev (2020)
*T.flavidobadia*	Yuan 11044	—	MK446364	—	—	China	Yuan et al. (2020)
*T.flavidobadia*	Yuan 11061	—	MK446365	—	—	China	Yuan et al. (2020)
*T.fuscocinerea*	TU108229	GU214810	—	—	—	Estonia	Unpublished
*T.fuscocrustosa*	Yuan 11420	MK211713	MK446367	—	—	China	Yuan et al. (2020)
*T.fuscocrustosa*	Yuan 11399	MK211712	MK446366	—	—	China	Yuan et al. (2020)
*T.fuscofarinosa*	Yuan 12142	MK211715	MK446369	—	—	China	Yuan et al. (2020)
*T.fuscofarinosa*	Yuan 12125	MK211714	MK446368	—	—	China	Yuan et al. (2020)
*T.fuscogranulosa*	Yuan 10723	—	MK446370	—	—	China	Yuan et al. (2020)
*T.fuscogranulosa*	Yuan 10725	—	MK446371	—	—	China	Yuan et al. (2020)
*T.fuscopelliculosa*	Yuan 11316	MK211717	—	—	—	China	Yuan et al. (2020)
*T.fuscopelliculosa*	Yuan 11305	MK211716	MK446372	—	—	China	Yuan et al. (2020)
*T.galzinii*	TAA166821	AF272932	—	—	—	Estonia	Kõljalg et al. (2001)
*T.galzinii*	TAA149734	AF272928	—	—	—	Estonia	Kõljalg et al. (2001)
*T.globosa*	AMC122	OP413006	—	—	—	USA	Unpublished
*T.globosa*	Yuan 11603	—	MN684328	—	—	Finland	Lu et al. (2018)
*T.globospora*	Yuan 10668	—	MK446374	—	—	China	Yuan et al. (2020)
*T.globospora*	Yuan 10748	—	MK446375	—	—	China	Yuan et al. (2020)
*T.gloeocystidiata*	Yuan 11200	—	MK446377	—	—	China	Yuan et al. (2020)
*T.gloeocystidiata*	Yuan 11171	—	MK446376	—	—	China	Yuan et al. (2020)
*T.griseocastanea*	Yuan 11401	—	MK446378	—	—	China	Yuan et al. (2020)
*T.griseocastanea*	Yuan 11409	—	MK446379	—	—	China	Yuan et al. (2020)
*T.griseofusca*	Yuan 11104	—	MK446381	—	—	China	Yuan et al. (2020)
*T.griseofusca*	Yuan 11094	—	MK446380	—	—	China	Yuan et al. (2020)
*T.griseomarginata*	Yuan 11458	MK211720	MK446382	—	—	China	Yuan et al. (2020)
*T.griseomarginata*	Yuan 11468	MK211721	MK446383	—	—	China	Yuan et al. (2020)
*T.guiyangensis*	Yuan 18281	PP479645	PP486306	—	—	China	Zhu et al. (2024)
*T.guiyangensis*	Yuan 18256	PP479643	PP486300	—	—	China	Zhu et al. (2024)
*T.guineensis*	M SYN 2331	NR_119955	—	—	—	Guinea	Yorou et al. (2012b)
*T.guineensis*	SYN 2331	JF520432	—	—	—	Guinea	Yorou et al. (2012b)
*T.hjortstamiana*	TU103641	NR_121290	—	—	—	Seychelles	Suvi et al. (2010)
*T.inconspicua*	Yuan 11107	—	MK446385	—	—	China	Yuan et al. (2020)
*T.inconspicua*	Yuan 11060	—	MK446384	—	—	China	Yuan et al. (2020)
*T.incrustata*	Yuan 12189	MK211723	MK446387	—	—	China	Yuan et al. (2020)
*T.incrustata*	Yuan 11158	MK211722	MK446386	—	—	China	Yuan et al. (2020)
*T.interrupta*	Yuan 10775	—	MK446388	—	—	China	Yuan et al. (2020)
*T.interrupta*	Yuan 11203	—	MK446389	—	—	China	Yuan et al. (2020)
*T.intsiae*	TAA195077	AM412296	—	—	—	Estonia	Tedersoo et al. (2007)
*T.intsiae*	TU105130	NR_121286	—	—	—	Seychelles	Suvi et al. (2010)
*T.lapida*	LE F-332369	MT981496	—	—	—	Russia	Ivanushenko and Volobuev (2020)
*T.lapida*	PN_2Bb_I	JQ724049	—	—	—	Poland	Hrynkiewicz et al. (2012)
*T.larssoniana*	TU103690	AM412294	—	—	—	Estonia	Tedersoo et al. (2007)
*T.larssoniana*	TU105082	NR_119738	—	—	—	Estonia	Suvi et al. (2010)
*T.lilacinogrisea*	NS74	DQ068972	—	—	—	Sweden	Menkis et al. (2005)
*T.lilacinogrisea*	AR1119	JX630832	—	—	—	USA	Timling et al. (2012)
*T.longechinulata*	Yuan 11979	MK211726	MK446393	—	—	China	Yuan et al. (2020)
*T.longechinulata*	Yuan 12083	MK211727	MK446394	—	—	China	Yuan et al. (2020)
*T.longiaculeifera*	Yuan 10744	—	MK446391	—	—	China	Yuan et al. (2020)
*T.longiaculeifera*	Yuan 11119	—	MK446392	—	—	China	Yuan et al. (2020)
*T.longiechinula*	Yuan 12687	—	MK850201	—	—	Vietnam	Lu et al. (2022)
*T.longiechinula*	Yuan 12720	—	MK850200	—	—	Vietnam	Lu et al. (2022)
*T.longisterigmata*	IFP 19181	NR_161037	—	—	—	Finland	Lu et al. (2018)
*T.maroana*	M SYN 878	NR_119636	—	—	—	Benin	Yorou and Agerer (2008)
*T.maroana*	SYN 878	EF507250	—	—	—	Benin	Yorou and Agerer (2008)
*T.megaspora*	Yuan 11326	—	MK446395	—	—	China	Yuan et al. (2020)
*T.megaspora*	Yuan 11472	—	MK446396	—	—	China	Yuan et al. (2020)
*T.muricata*	O-F256712	MT146462	—	—	—	Sweden	Svantesson et al. (2021)
*T.muricata*	O-F256713	MT146461	—	—	—	Sweden	Svantesson et al. (2021)
*T.nitellina*	src675	DQ974778	—	—	—	USA	Smith et al. (2007)
*T.olivacea*	Yuan 11043	—	MK446397	—	—	China	Yuan et al. (2020)
*T.olivacea*	Yuan 11139	—	MK446398	—	—	China	Yuan et al. (2020)
***T.olivaceobasidiosa***	**CLZhao 14051**	**PP810228**	—	**PQ060163**	**PQ156137**	**China**	**Present study**
***T.olivaceobasidiosa***	**CLZhao 14056**	**PP810229**	**PP809698**	**PQ060164**	**PQ156138**	**China**	**Present study**
*T.olivaceobrunnea*	Yuan 12148	—	MK446400	—	—	China	Yuan et al. (2020)
*T.olivaceobrunnea*	Yuan 11194	—	MK446399	—	—	China	Yuan et al. (2020)
*T.olivaceomarginata*	Yuan 18268	PP479639	PP486303	—	—	China	Zhu et al. (2024)
*T.olivaceomarginata*	Dai 25782	PP479640	—	—	—	China	Zhu et al. (2024)
*T.pallidobrunnea*	Yuan 11493	MK211731	MK446402	—	—	China	Yuan et al. (2020)
*T.pallidobrunnea*	Yuan 11481	MK211730	MK446401	—	—	China	Yuan et al. (2020)
*T.pallidocastanea*	Yuan 11416	—	MN684323	—	—	China	Lu et al. (2018)
*T.pallidocastanea*	Yuan 12034	—	MN684324	—	—	China	Lu et al. (2018)
*T.pallidomarginata*	Yuan 11474	MK211733	MK446404	—	—	China	Yuan et al. (2020)
*T.pallidomarginata*	Yuan 11404	MK211732	MK446403	—	—	China	Yuan et al. (2020)
*T.parmastoana*	NAN13	MN075506	—	—	—	Thailand	Unpublished
*T.parmastoana*	TU 103582	NR_121289	—	—	—	USA	Tedersoo et al. (2007)
*T.parvispora*	Yuan 11196	—	MK446406	—	—	China	Yuan et al. (2020)
*T.parvispora*	Yuan 11144	—	MK446405	—	—	China	Yuan et al. (2020)
*T.patagonica*	BAFC52372	NR_159018	—	—	—	Argentina	Kuhar et al. (2016)
*T.patagonica*	LR-24	MT366710	—	—	—	USA	Unpublished
*T.pertenuis*	Yuan 11064	—	MK446407	—	—	China	Yuan et al. (2020)
*T.pertenuis*	Yuan 11131	—	MK446408	—	—	China	Yuan et al. (2020)
*T.pileocystidiata*	TU105068	NR_119739	—	—	—	Estonia	Suvi et al. (2010)
*T.pileocystidiata*	TU105054	FM955845	—	—	—	Estonia	Suvi et al. (2010)
*T.pilosa*	TU124067	MT146459	MT554521	—	—	Sweden	Svantesson et al. (2021)
*T.pilosa*	TU124234	MT146458	—	—	—	Sweden	Svantesson et al. (2021)
*T.pisoniae*	TU103671	NR_121358	—	—	—	USA	Suvi et al. (2010)
*T.pisoniae*	TU103655	FN185986	—	—	—	Argentina	Kuhar et al. (2016)
*T.pulvinulata*	BAFC52370	NR_159017	—	—	—	Argentina	Kuhar et al. (2016)
*T.qingyuanensis*	Yuan 10616	—	MK446409	—	—	China	Yuan et al. (2020)
*T.qingyuanensis*	Yuan 11109	—	MK446410	—	—	China	Yuan et al. (2020)
*T.rotundata*	Yuan 18269	PP479641	PP486304	—	—	China	Zhu et al. (2024)
*T.rotundata*	Yuan 18273	PP479642	PP486305	—	—	China	Zhu et al. (2024)
*T.segregata*	Yuan 10650	—	MK446411	—	—	China	Yuan et al. (2020)
*T.segregata*	Yuan 11256	—	MK446412	—	—	China	Yuan et al. (2020)
*T.separata*	Yuan 10664	MK211737	MK850196	—	—	China	Yuan et al. (2020)
*T.separata*	Yuan 10654	MK211736	MK850197	—	—	China	Yuan et al. (2020)
*T.stipitata*	Yuan 11160	—	MK446413	—	—	China	Yuan et al. (2020)
*T.storea*	Yuan 10749	—	MK446416	—	—	China	Yuan et al. (2020)
*T.storea*	Yuan 10623	—	MK446415	—	—	China	Yuan et al. (2020)
*T.stuposa*	IB2005314	EF644117	—	—	—	Australia	Krpata et al. (2008)
*T.subclavigera*	O-F256725	MT146460	—	—	—	Sweden	Svantesson et al. (2021)
*T.subtestacea*	FFP816	JQ711878	—	—	—	Canada	Jones et al. (2012)
*T.subtestacea*	FR-F10	MW546519	—	—	—	South Korea	Unpublished
*T.tedersooi*	TU103663	NR_121359	—	—	—	Estonia	Suvi et al. (2010)
*T.tedersooi*	TU103664	FN185989	—	—	—	Estonia	Suvi et al. (2010)
*T.tenuirhizomorpha*	Yuan 12059	MG799185	MN684327	—	—	China	Lu et al. (2018)
*T.tenuissima*	FK14070	KT032087	—	—	—	Argentina	Kuhar et al. (2016)
*T.tenuissima*	BAFC52369	NR_159016	—	—	—	USA	Kõljalg et al. (2001)
*T.terrestris*	EL9897	AF272901	—	—	—	Estonia	Kõljalg et al. (2001)
*T.terrestris*	TAA159557	AF272911	—	—	—	Estonia	Kõljalg et al. (2001)
***T.velutina***	**CLZhao 25474**	**PP645440**	**PP809700**	**PQ060166**		**China**	**Present study**
*T.verruculata*	Yuan 12684	—	MN684331	—	—	China	Lu et al. (2022)
*T.verruculata*	Yuan 12680	—	MN684332	—	—	China	Lu et al. (2022)
*T.viridula*	MTB37	MN947374	—	—	—	Estonia	Kõljalg et al. (2001)
***T.wumenshanensis***	**CLZhao 33775**	**PP810230**	**PP809699**	**PQ060165**	—	**China**	**Present study**
***T.yunnanensis***	**CLZhao 32532**	**PP810231**	—	—	—	**China**	**Present study**
*Tomentellopsisrosannae*	MES-3338	MT366690	—	—	—	Chile	Kuhar et al. (2022)
*T.submollis*	RS-22498	AJ410774	—	—	—	Finland	Kuhar et al. (2022)
*T.submollis*	P24-F	AM086447	—	—	—	Norway	Kuhar et al. (2022)
*T.zygodesmoides*	JS-27216	AJ410759	—	—	—	Norway	Kuhar et al. (2022)
*T.zygodesmoides*	KHL-8653	AJ410761	—	—	—	Norway	Kuhar et al. (2022)

**Figure 1.**
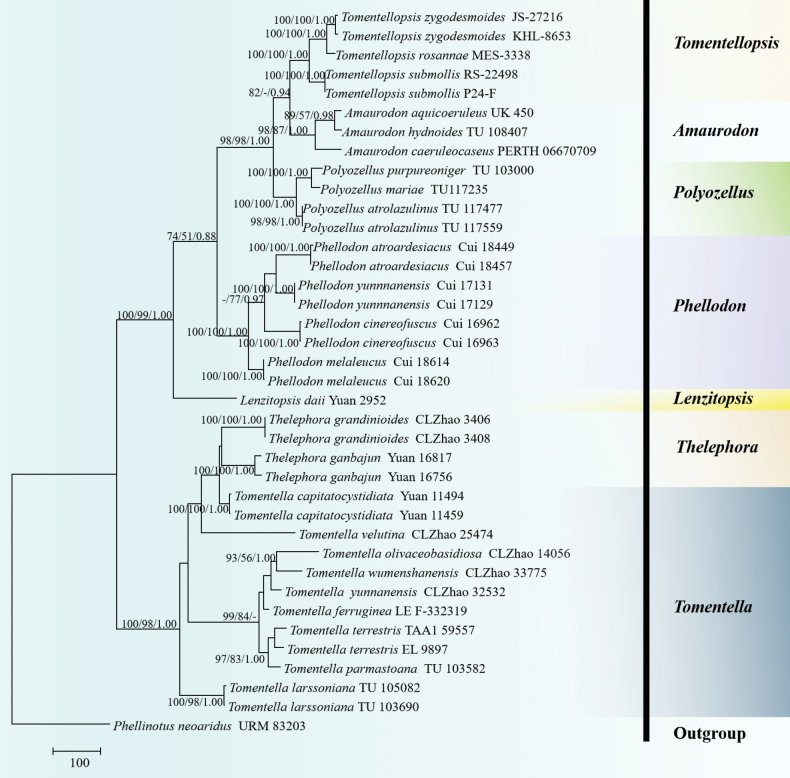
Maximum parsimony strict consensus tree illustrating the phylogeny of *Tomentella* and related genera in the family Thelephoraceae, based on ITS+nLSU+mtSSU+RPB2 sequences. Branches are labelled with maximum likelihood bootstrap values ≥ 70%, parsimony bootstrap values ≥ 50% and Bayesian posterior probabilities ≥ 0.95, respectively.

**Figure 2.**
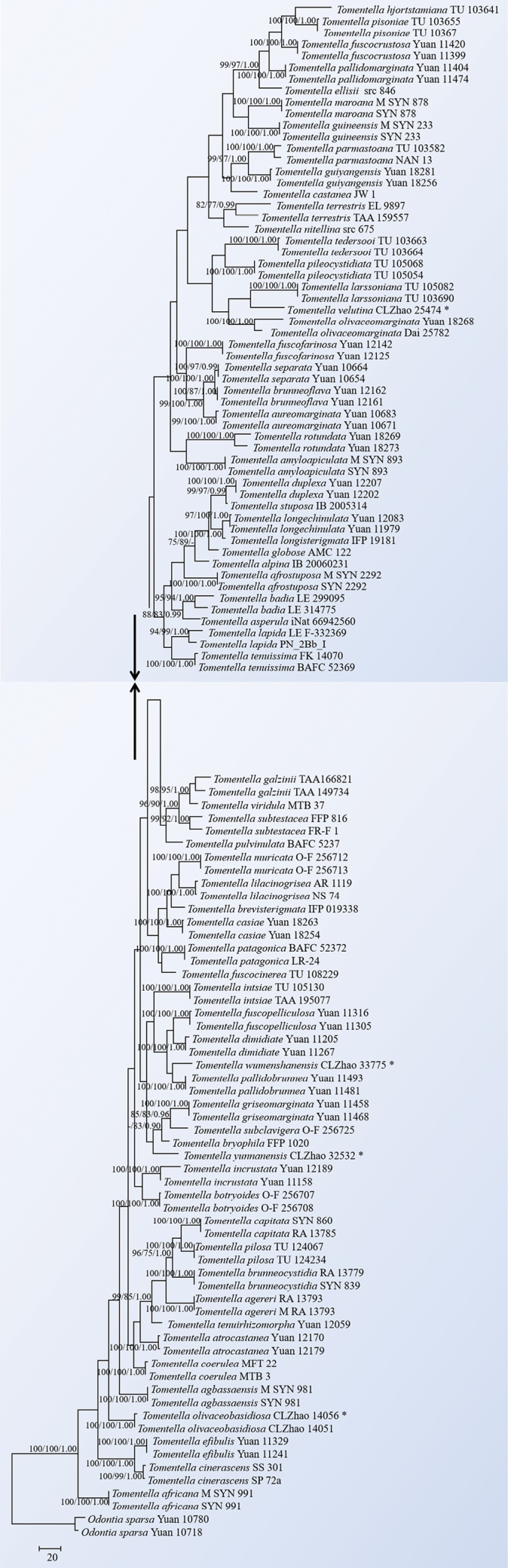
Maximum parsimony strict consensus tree illustrating the phylogeny of the three new species and related species in *Tomentella*, based on ITS sequences. Branches are labelled with maximum likelihood bootstrap values ≥ 70%, parsimony bootstrap values ≥ 50% and Bayesian posterior probabilities ≥ 0.95, respectively. The type species are marked with *.

The sequences were aligned in MAFFT v. 7 (Katoh et al. 2019) using the G-INS-i strategy. The alignment was adjusted manually using AliView v. 1.27 (Larsson 2014). The sequence alignments were deposited in TreeBase (ID 31627). Sequences of *Phellinotusneoaridus* Drechsler-Santos & Robledo. Parmasto retrieved from GenBank was used as an outgroup in the ITS+nLSU+mtSSU+RPB2 analysis (Fig. 1; Salvador-Montoya et al. (2022)). The sequence alignments were deposited in TreeBase (ID 31628). Sequences of *Odontiasparsa* Yuan Yuan, Y.C. Dai & H.S. Yuan retrieved from GenBank were used as the outgroups in the ITS+nLSU analysis (Fig. 2; Yuan et al. (2018)).

Maximum parsimony (MP), Maximum Likelihood (ML) and Bayesian Inference (BI) analyses were applied to the combined three datasets following a previous study (Zhao et al. 2023a). All characters were equally weighted and gaps were treated as missing data. Trees were inferred using the heuristic search option with TBR branch swapping and 1,000 random sequence additions. Max-trees were set to 5,000, branches of zero length were collapsed and all parsimonious trees were saved. Clade robustness was assessed using bootstrap (BT) analysis with 1,000 pseudo replicates (Felsenstein 1985). Descriptive tree statistics-tree length (TL), composite consistency index (CI), composite retention index (RI), composite rescaled consistency index (RC) and composite homoplasy index (HI) - were calculated for each maximum parsimonious tree generated. The combined dataset was also analysed using Maximum Likelihood (ML) in RAxML-HPC2 through the CIPRES Science Gateway (Miller et al. 2006). Branch support (BS) for the ML analysis was determined by 1000 bootstrap pseudo replicates.

MrModelTest 2.3 (Nylander 2004) was used to determine the best-ﬁt evolution model for each dataset for Bayesian inference (BI), which was performed using MrBayes 3.2.7a with a best model of DNA substitution and a gamma distribution rate variation across sites (Ronquist et al. 2012). A total of four Markov chains were run for two runs from random starting trees for 2 million generations for ITS+nLSU+mtSSU+RPB2 (Fig. 1) and 12 million generations for ITS+nLSU (Fig. 2) with trees and parameters sampled every 1,000 generations. The ﬁrst quarter of all of the generations were discarded as burn-ins. A majority rule consensus tree was computed from the remaining trees. Branches were considered as significantly supported if they received a maximum likelihood bootstrap support value (BS) of ≥ 70%, a maximum parsimony bootstrap support value (BT) of ≥ 70% or a Bayesian posterior probability (BPP) of ≥ 0.95.

## ﻿Results

### ﻿Molecular phylogeny

The ITS+nLSU+mtSSU+RPB2 dataset (Fig. 1) comprised sequences from 38 fungal specimens representing 26 taxa. The dataset had an aligned length of 6,608 characters, of which 5,402 characters were constant, 318 were variable and parsimony-uninformative and 888 were parsimony-informative. Maximum parsimony analysis yielded one equally parsimonious tree (TL = 2,559, CI = 0.6444, HI = 0.3566, RI = 0.7784 and RC = 0.5016). The best model of nucleotide evolution for the ITS+nLSU+mtSSU+RPB2 dataset estimated and applied in the Bayesian analysis was GTR+I+G. Bayesian analysis and ML analysis resulted in a similar topology to the MP analysis. The Bayesian analysis had an average standard deviation of split frequencies = 0.009434 (BI) and the effective sample size (ESS) across the two runs is double the average ESS (avg. ESS) = 950. The phylogram, based on the ITS+nLSU+mtSSU+RPB2 rDNA gene regions (Fig. 1), included seven genera within the family Thelephoraceae (Thelephorales), including *Amaurodon*, *Lenzitopsis* Malençon & Bertault, *Phellodon* P. Karst, *Polyozellus* Murrill, *Thelephora* Ehrh. ex Willd., *Tomentella* and *Tomentellopsis*, in which four new species were nested into the genus *Tomentella*.

The ITS+nLSU dataset (Fig. 2) comprised sequences from 115 fungal specimens representing 69 taxa. The dataset had an aligned length of 2117 characters, of which 1664 characters were constant, 71 were variable and parsimony-uninformative and 340 were parsimony-informative. Maximum parsimony analysis yielded one equally parsimonious tree (TL = 2,501, CI = 0.2735, HI = 0.7265, RI = 0.5884 and RC = 0.1609). The best model of nucleotide evolution for the ITS dataset estimated and applied in the Bayesian analysis was GTR+I+G. Bayesian analysis and ML analysis resulted in a topology similar to that of the MP analysis. The Bayesian analysis had an average standard deviation of split frequencies = 0.009654 (BI) and the effective sample size (ESS) across the two runs is double the average ESS (avg. ESS) = 360. The phylogenetic tree (Fig. 2) showed that the new species *Tomentellaolivaceobasidiosa* formed a monophyletic lineage in the ITS+nLSU phylogetic tree. Furthermore, the new species *T.velutina* was sister to *T.larssoniana* Suvi & Kõljalg. The new taxon, *T.wumenshanensis* was sister to *T.pallidobrunnea* H.S. Yuan, X. Lu & Y.C. Dai. Moreover, the new species *T.yunnanensis* was grouped closely with three taxa *T.bryophila* (Pers.) M.J. Larsen, *T.griseomarginata* H.S. Yuan, X. Lu & Y.C. Dai and *T.subclavigera* Litsch.

### ﻿Taxonomy

#### ﻿ Tomentella olivaceobasidiosa

Taxon classificationFungiThelephoralesThelephoraceae

X.J. Zhang & C.L. Zhao sp. nov.

2597ABD5-CF56-59C7-B535-856319CD852F

 854694

Figs 3 , 4

##### Holotype.

China • Yunnan Province, Kunming, Panlong District, Yeyahu Forest Park, 25°13'N, 102°87'E, altitude 2125 m, on the angiosperm trunk, leg. C.L. Zhao, 30 September 2019, CLZhao 14056 (SWFC).

**Figure 3.**
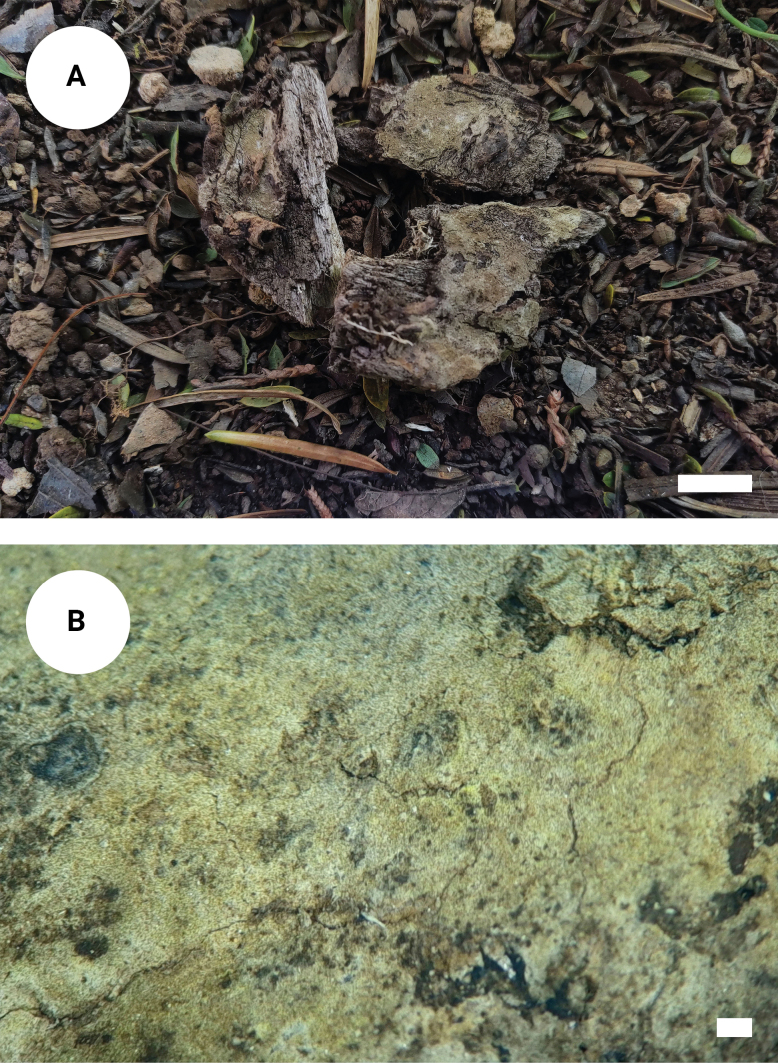
*Tomentellaolivaceobasidiosa* (holotype, CLZhao 14056): basidiomata on the substrate (**A**), macroscopic characteristics of hymenophore (**B**). Scale bars: 1 cm (**A**); 1 mm (**B**).

##### Etymology.

*Olivaceobasidiosa* (Lat.): refers to the olivaceous basidiomata.

**Figure 4.**
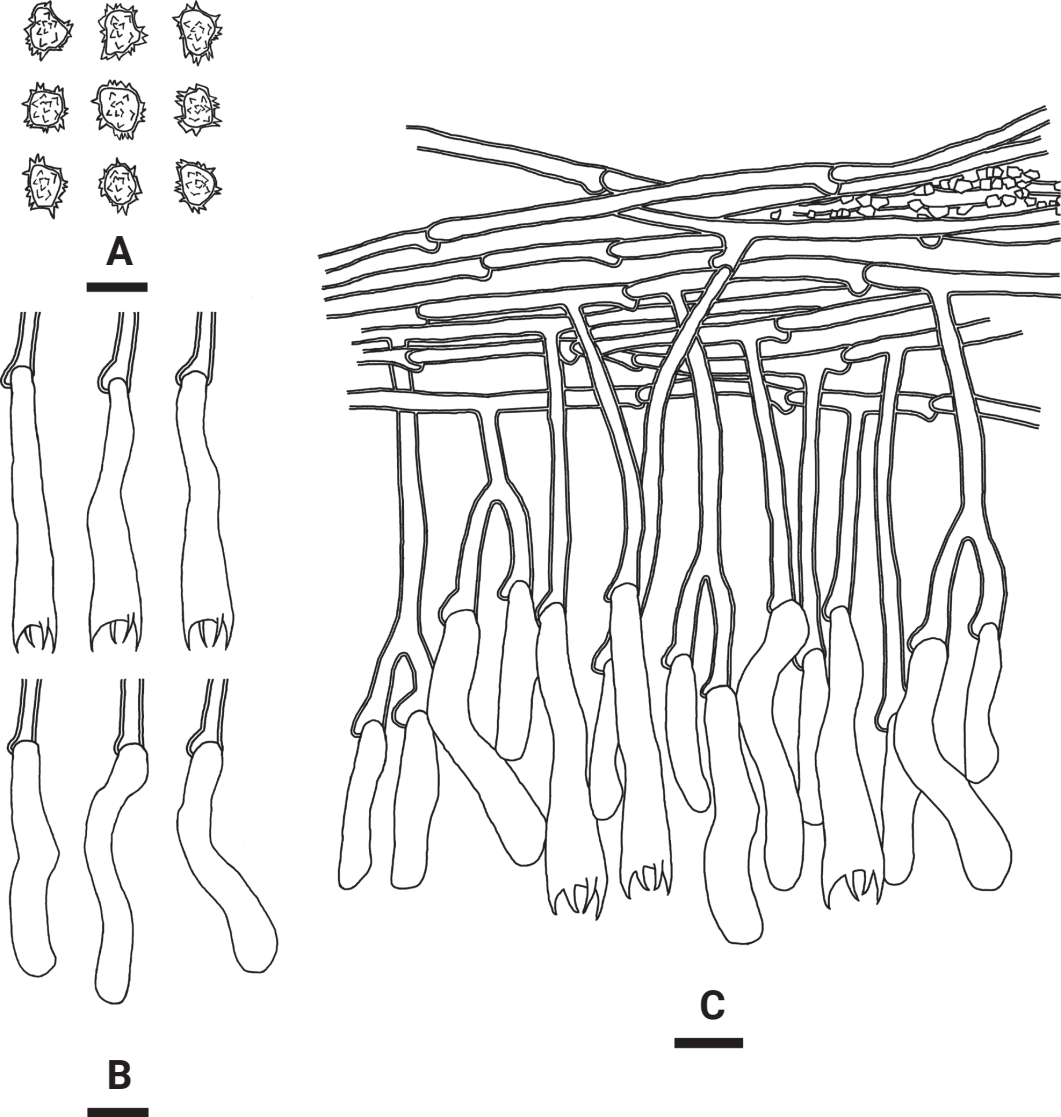
Microscopic structures of *Tomentellaolivaceobasidiosa* (holotype, CLZhao 14056): basidiospores (**A**), basidia and basidioles (**B**), a section of the hymenium (**C**). Scale bars: 10 µm (**A–C**).

##### Description.

Basidiomata annual, resupinate, separable from the substrate, arachnoid, without odour or taste when fresh, and up to 3 cm long, 2.5 cm wide, 0.3–0.6 mm thick. Hymenial surface smooth, slightly olivaceous when fresh, olivaceous to slightly brown upon drying. Sterile margin narrow, olivaceous, up to 1 mm.

***Hyphal system***: Monomitic; generative hyphae with clamp connections, pale brown, slightly thick-walled, moderately branched, interwoven, 3.5–5 µm in diameter. IKI–, CB–; brown-black to black in KOH.

***Hymenium***: Cystidia and cystidioles absent. Presence of crystals amongst generative hyphae. Basidia clavate, with 4 sterigmata and a basal clamp connection, 39–48.5 × 7–8.5 µm, basidiole clavate, slightly smaller than basidia.

***Spores***: Basidiospores subglobose to globose, nodulose to verrucose, yellowish-brown, thick-walled, IKI–, CB–, (6.5–)7–9(–9.5) × (5–)6–7.5(–8.5) µm, L = 8.4 µm, W = 7 µm, Q = 1.20–1.23 (n = 60/2).

Additional specimen examined (paratype): China. Yunnan Province, Kunming, Panlong District, Yeyahu Forest Park, GPS coordinates: 25°13'N, 102°87'E, elev. 2125 m, on the angiosperm trunk, leg. C.L. Zhao, 30 September 2019, CLZhao 14051 (SWFC).

#### ﻿ Tomentella velutina

Taxon classificationFungiThelephoralesThelephoraceae

X.J. Zhang & C.L. Zhao sp. nov.

CBE88FC5-7A8C-5EE3-A169-08C3C0B6C61B

 854695

Figs 5 , 6

##### Holotype.

China • Yunnan Province, Lincang, Fengqing County, 24°66'N, 100°19'E, altitude 2060 m, on the fallen branch of angiosperm, leg. C.L. Zhao, 22 October 2022, CLZhao 25474 (SWFC).

**Figure 5.**
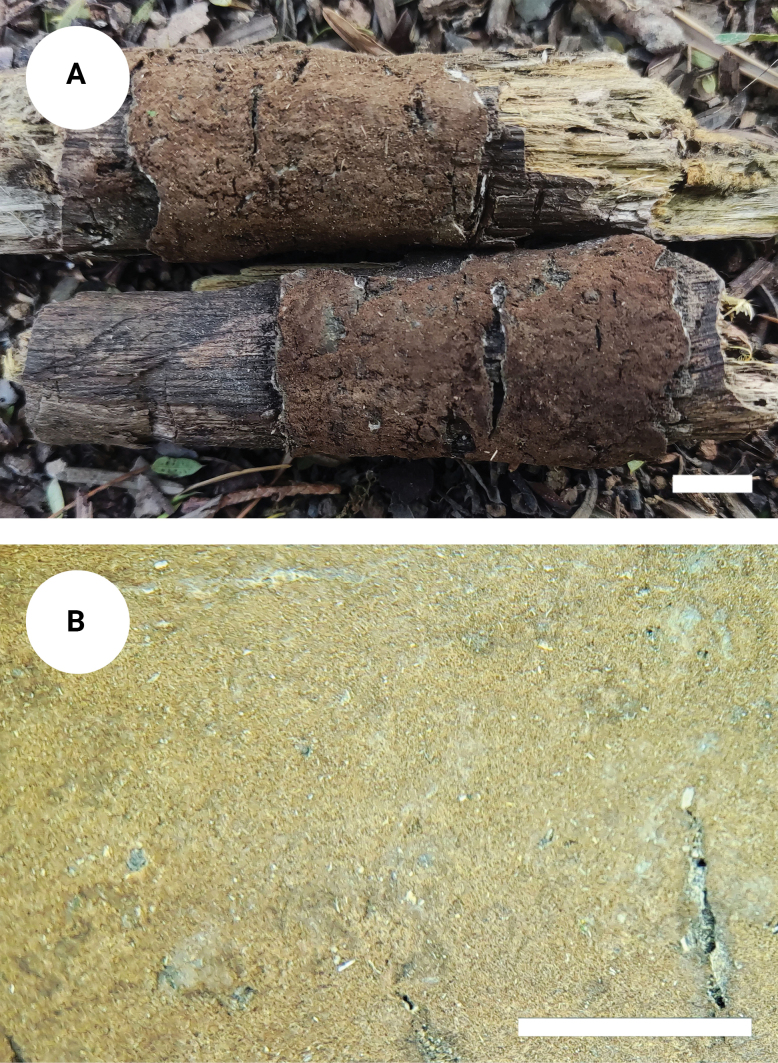
*Tomentellavelutina* (holotype, CLZhao 25474): basidiomata on the substrate (**A**), macroscopic characteristics of hymenophore (**B**). Scale bars: 1 cm (**A**); 1 mm (**B**).

##### Etymology.

*Velutina* (Lat.): refers to the velvety hymenophore of the type specimen.

**Figure 6.**
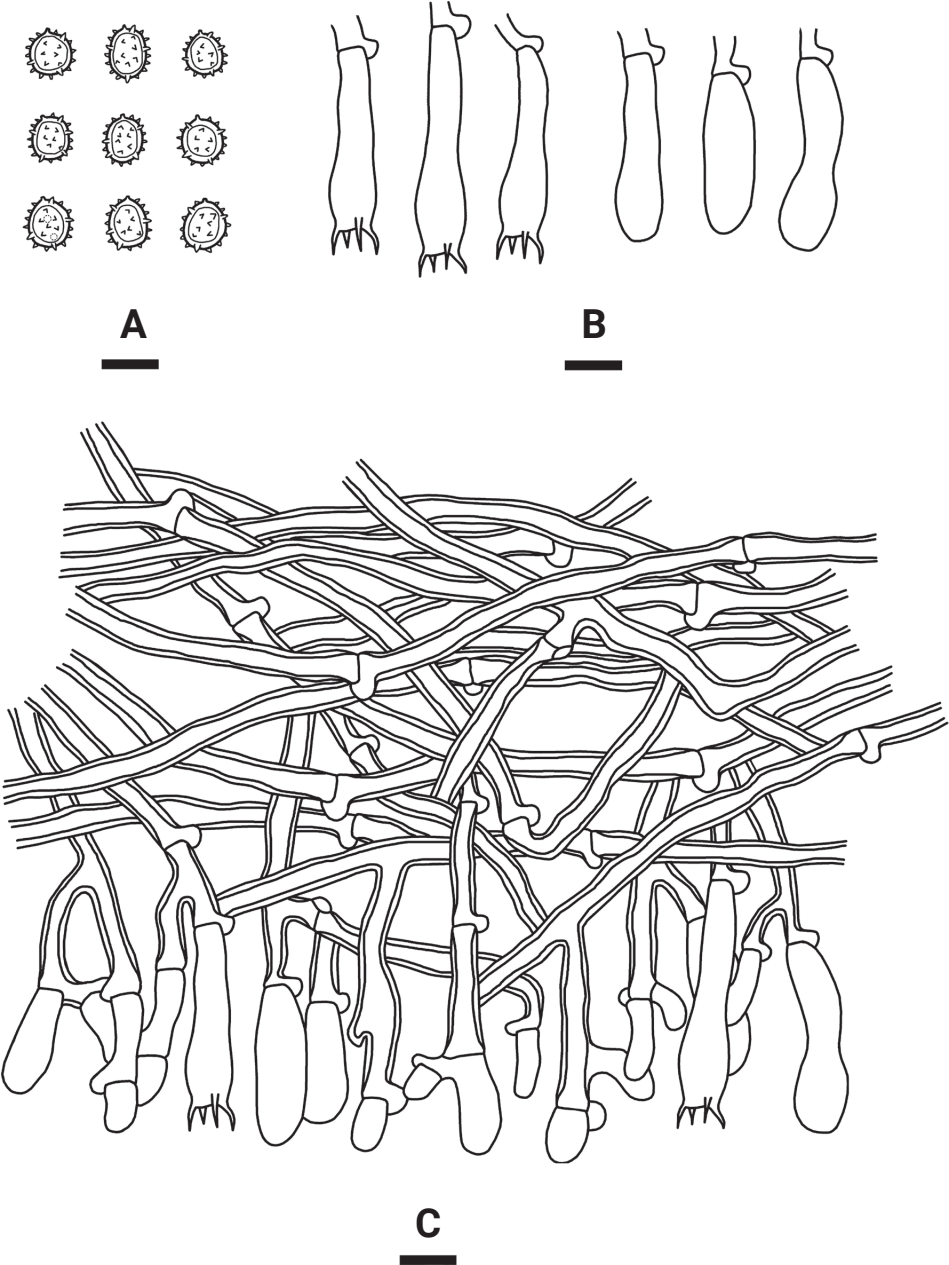
Microscopic structures of *Tomentellavelutina* (holotype, CLZhao 25474): basidiospores (**A**), basidia and basidioles (**B**), a section of the hymenium (**C**). Scale bars: 10 µm (**A–C**).

##### Description.

Basidiomata annual, resupinate, adnate, cotton to floccose, without odour and taste when fresh and up to 4.5 cm long, 3 cm wide, 0.3–0.5 mm thick. Hymenial surface cotton to floccose, fawn to reddish brown when fresh, turn reddish brown to vinaceous brown when dry. Sterile margin narrow, fawn to reddish brown, up to 2 mm.

***Hyphal system***: Monomitic; generative hyphae with clamp connections, yellowish-brown, slightly thick-walled, 3–7 mm in diameter, IKI–, CB–, brown-black to black in KOH.

***Hymenium***: Cystidia and cystidioles absent. Basidia clavate, colourless, with 4 sterigmata and a basal clamp connection 36–42.5 × 7–8 µm.

***Spores***: Basidiospores broadly ellipsoid, yellowish-brown, thick-walled, ornamented, with 1–2 oil drops, CB–, IKI–, 7–9 × (5.5–)6–7.5 µm, L = 7.82 µm, W = 6.71 µm, Q = 1.16 (n = 30/1).

#### ﻿ Tomentella wumenshanensis

Taxon classificationFungiThelephoralesThelephoraceae

X.J. Zhang & C.L. Zhao sp. nov.

9637A8E6-2F66-5522-A854-E9171691F334

 854696

Figs 7 , 8

##### Holotype.

China • Yunnan Province, Zhaotong, Wumengshan National Nature Reserve, 27°33'N, 103°72'E, altitude 2300 m, on the fallen branch of angiosperm, leg. C.L. Zhao, 21 September 2023, CLZhao 33775 (SWFC).

**Figure 7.**
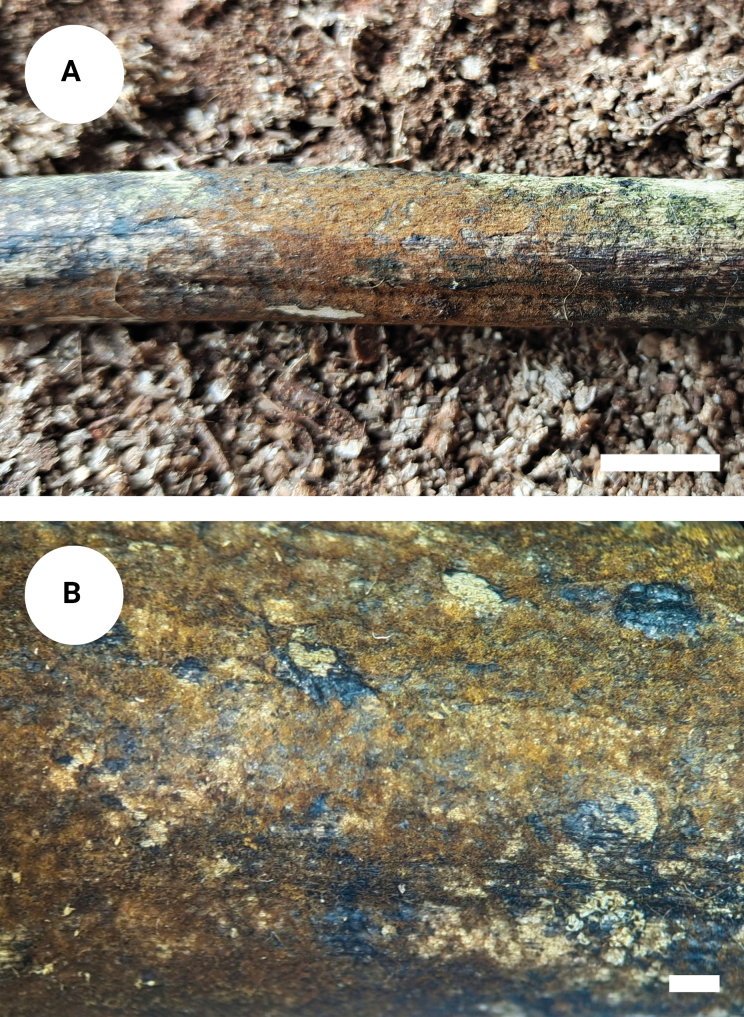
*Tomentellawumenshanensis* (holotype, CLZhao 33775): basidiomata on the substrate (**A**), macroscopic characteristics of hymenophore (**B**). Scale bars: 1 cm (**A**); 1 mm (**B**).

##### Etymology.

*Wumenshanensis* (Lat.): refers to the type locality “Wumengshan National Nature Reserve”.

**Figure 8.**
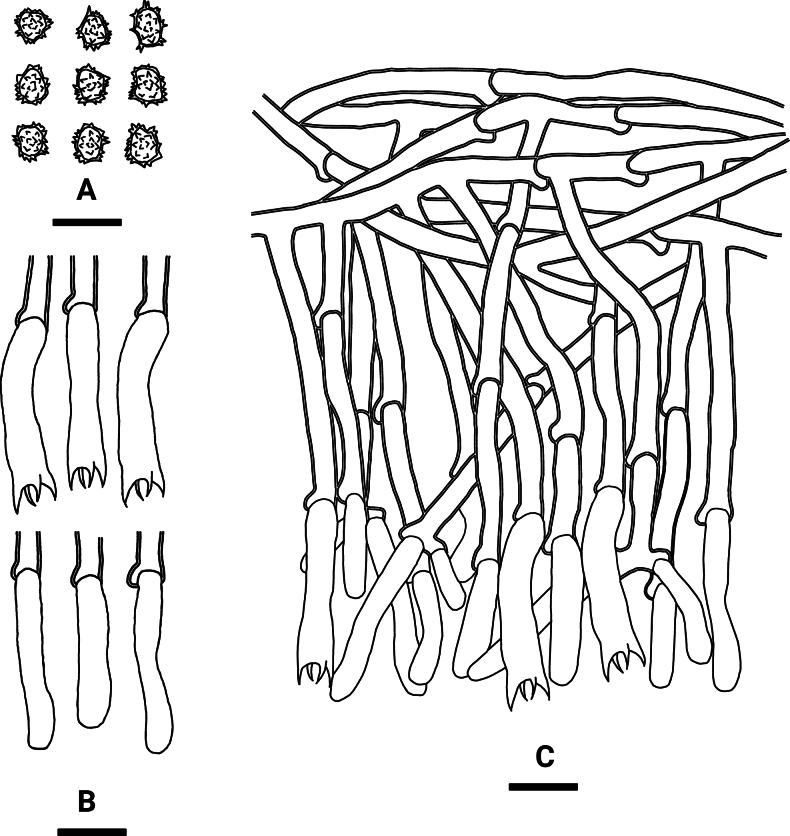
Microscopic structures of *Tomentellawumenshanensis* (holotype, CLZhao 33775): basidiospores (**A**), basidia and basidioles (**B**), a section of the hymenium (**C**). Scale bars: 10 µm (**A–C**).

##### Description.

Basidiomata annual, resupinate, membranaceous, without odour or taste when fresh, up to 15 cm long, 2 cm wide, 0.1–0.2 mm thick. Hymenial surface smooth, yellowish-brown to orange brown when dry. Sterile margin narrow, yellowish-brown, up to 1 mm.

***Hyphal system***: Monomitic; generative hyphae with clamp connections, yellowish-brown, slightly thick-walled, moderately branched, interwoven, 5–8 µm in diameter, IKI–, CB–; brown-black to black in KOH.

***Hymenium***: Cystidia and cystidioles absent. Basidia barrel-shaped to slightly clavate, with 4 sterigmata and a basal clamp connection, 25–28 × 5.5–8.5 µm; basidioles dominant, slightly smaller than basidia.

***Spores***: Basidiospores subglobose to globose, nodulose to verrucose, yellowish-brown, thick-walled, IKI–, CB–, (7–) 7.5–9.5(–10) × 6–8(–8.5) µm, L = 8.3 µm, W = 7 µm, Q = 1.19 (n = 30/1).

#### ﻿ Tomentella yunnanensis

Taxon classificationFungiThelephoralesThelephoraceae

X.J. Zhang & C.L. Zhao sp. nov.

1C57FFE9-9040-56AB-931D-0217CB6FB3D5

 854697

Figs 9 , 10

##### Holotype.

China • Yunnan Province, Zhaotong, Wumengshan National Nature Reserve, 27°33'N, 103°72'E, altitude 2300 m, on the fallen branch of angiosperm, leg. C.L. Zhao, 28 August 2023, CLZhao 32532 (SWFC).

**Figure 9.**
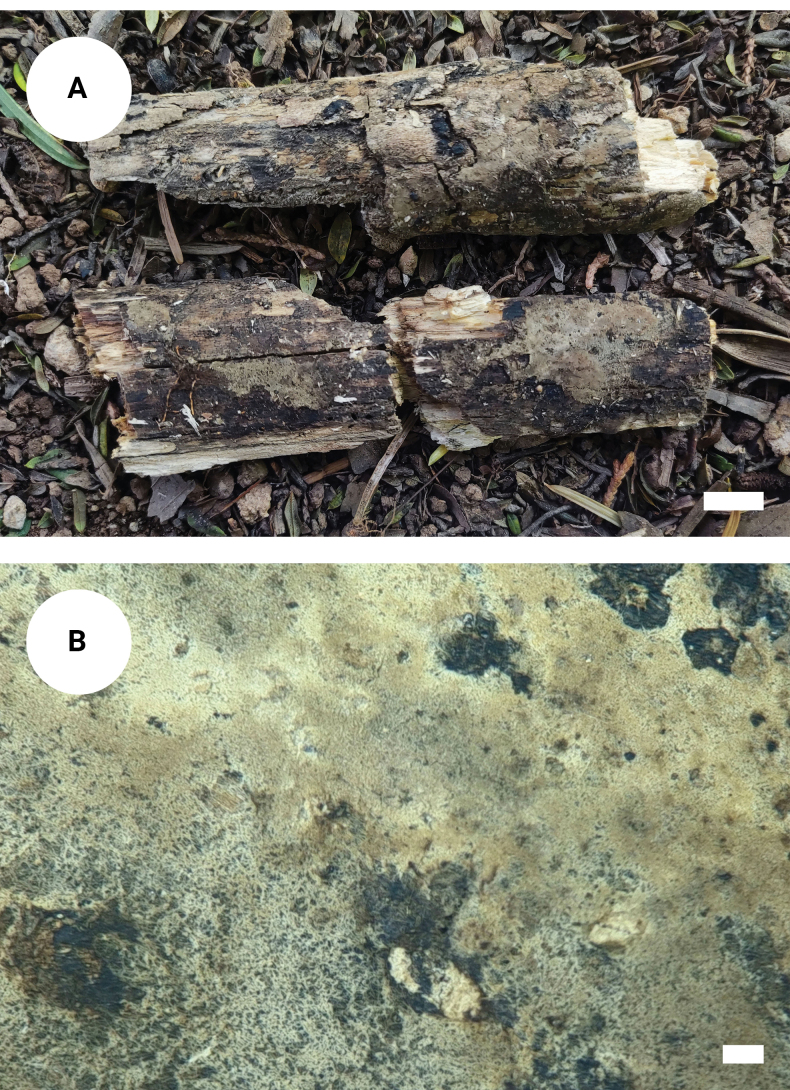
*Tomentellayunnanensis* (holotype, CLZhao 32532): basidiomata on the substrate (**A**), macroscopic characteristics of hymenophore (**B**). Scale bars: 1 cm (**A**); 1 mm (**B**).

##### Etymology.

*Yunnanensis* (Lat.): refers to the type locality “Yunnan Province”.

**Figure 10.**
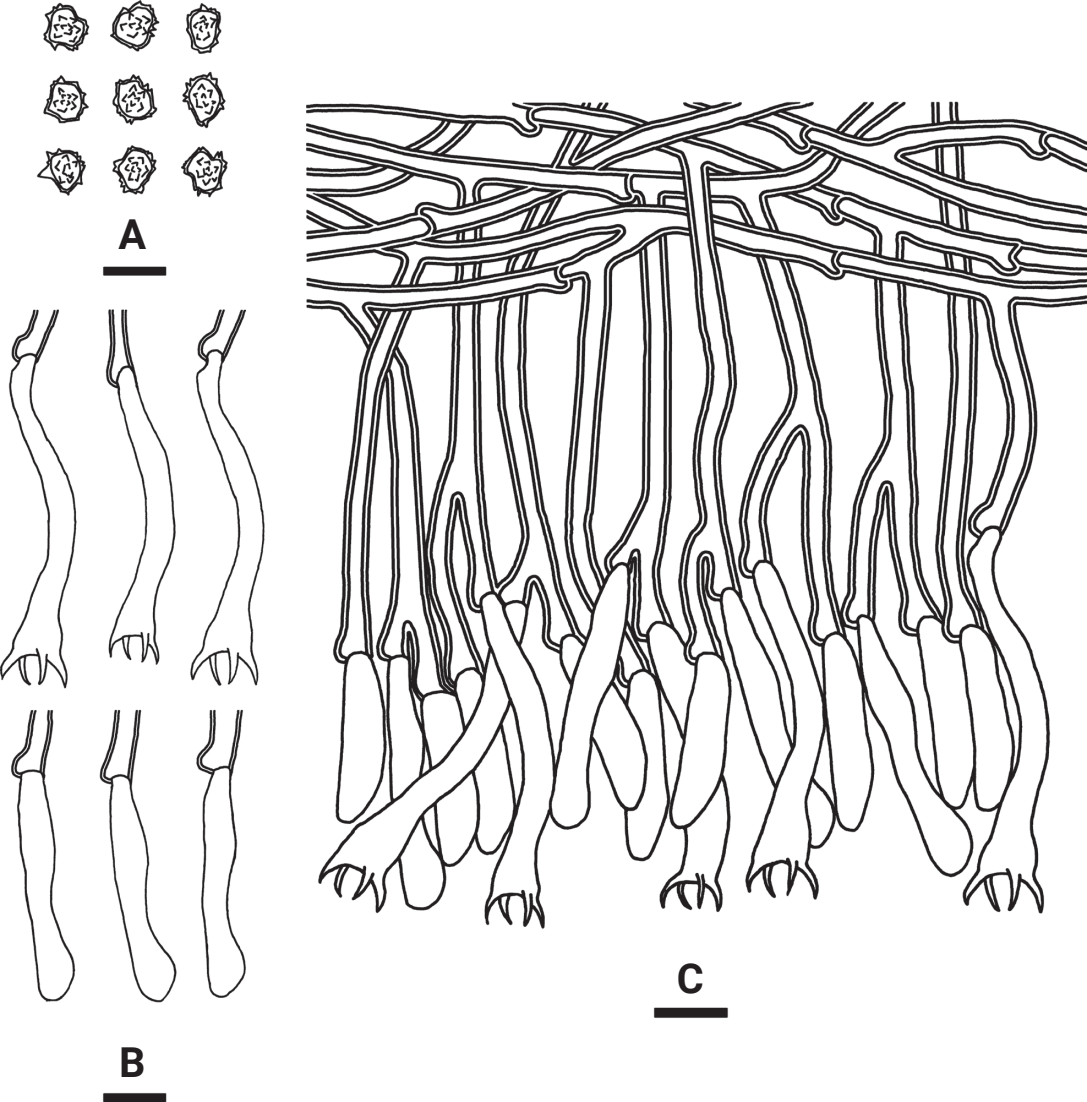
Microscopic structures of *Tomentellayunnanensis* (holotype, CLZhao 32532): basidiospores (**A**), basidia and basidioles (**B**), a section of the hymenium (**C**). Scale bars: 10 µm (**A–C**).

##### Description.

Basidiomata annual, resupinate, arachnoid, without odour or taste when fresh, up to 10 cm long, 3 cm wide, 0.2–0.3 mm thick. Hymenial surface smooth, slightly buff when fresh, buff to cinnamon buff when dry. Sterile margin narrow, cream to slightly buff, up to 1 mm.

***Hyphal system***: Monomitic; generative hyphae with clamp connections, pale brown, slightly thick-walled, branched, interwoven, 4–5 µm in diameter, IKI–, CB–; brown-black to black in KOH.

***Hymenium***: Cystidia and cystidioles absent. Basidia cylindrical to subclavate, with 4 sterigmata and a basal clamp connection, 46–57 × 7–9.5 µm; basidioles slightly smaller than basidia.

***Spores***: Basidiospores subglobose to globose, nodulose to verrucose, yellowish-brown, thick-walled, IKI–, CB–, 7–8.5(–9) × 5–8 µm, L = 8.13 µm, W = 6.72 µm, Q = 1.21 (n = 30/1).

## ﻿Discussion

Recently, many wood-inhabiting fungal taxa have been reported worldwide (Cui et al. 2019; Guan et al. 2023; Luo et al. 2024; Zhou et al. 2024) and, in the present study, four new species of the genus *Tomentella* are reported, based on a combination of morphological features and molecular evidence. The macroscopical and anatomical characteristics can well recognise all of them; *T.olivaceomarginata* is characterised by the olivaceous margin of the basidiomata and the presence of the clavate basidia measuring 39–48.5 × 7–8.5 µm. *Tomentellavelutina* can be recognised by having adnate cotton to floccose basidiomata and the presence of the clavate basidia and broadly ellipsoid basidiospores measuring 7–9 × 6–7.5 µm with 1–2 oil drops. *Tomentellawumenshanensis* is characterised by the membranaceous basidiomata having a tuberculate pileal surface hymenial and the presence of the barrel-shaped to slightly clavate basidia measuring 25–28 × 5.5–8.5 µm. *Tomentellayunnanensis* can be characterised by the typical of the arachnoid basidiomata having cylindrical to subclavate basidia measuring 46–57 × 7–9.5 µm.

Molecular phylogenetic analyses of the previous studies revealed that the taxa of both genera, *Thelephora* and *Tomentella* were non-monophyletic groups, in which they were intermixed in molecular phylogeny (Stalpers 1993; Kõljalg 1996; Larsson 2014; Ramírez-López et al. 2015; Vizzini et al. 2016; Li et al. 2020). In the present study, the four-genes (ITS+nLSU+mtSSU+RPB2) phylogenetic analysis provided an improved resolution at the family level and showed that the genera *Thelephora* and *Tomentella* grouped together, which is consistent with previous results (Stalpers 1993; Kõljalg et al. 2001; Yorou et al. 2008; Vizzini et al. 2016; Zmitrovich et al. 2018; Li et al. 2020), and four new species were nested into the genus *Tomentella*. The phylogenetic tree divided *Tomentella* into several distinct clades and most of the clades are consistent with the previous ITS phylogenetic analyses (Yuan et al. 2020; Lu et al. 2022). This study identifies and describes four new *Tomentella* species from China, based on morphological characteristics and phylogenetic analyses combining ITS+nLSU sequences (Fig. 2).

Phylogenetic analyses revealed that the new species *Tomentellaolivaceobasidiosa* formed a monophyletic lineage. Morphologically, *T.aureomarginata* is distinguishable from *T.olivaceobasidiosa* by having the pelliculose basidiomata with the golden brown to yellowish brown hymenium surface and smaller, slightly thick-walled basidiospores measuring 6.5–7 × 6–6.5 µm (Yuan et al. 2020). The species *Tomentellabrunneoflava* is distinct from *T.olivaceobasidiosa* by its brownish yellow hymenium surface and smaller basidia measuring 10–30 × 3–5 µm (Yuan et al. 2020). The species *T.separata* is delimited from *T.olivaceobasidiosa* by having the pelliculose basidiomata with the honey yellow to yellowish brown hymenium surface and narrower basidia measuring 15–55 × 3.5–6 µm (Yuan et al. 2020).

Phylogenetic analyses revealed that the species *Tomentellavelutina* was sister to *T.larssoniana*. However, morphologically, *T.larssoniana* is different from *T.velutina* by the grey or dark grey hymenophore, thin-walled subicular hyphae and wider basidia measuring 26–43 × 8.2–11 µm (Suvi et al. 2010). Morphologically, *Tomentellacasiae* H.S. Yuan & Y.Q. Zhu differs from *T.velutina* by the granulose, greyish to grey hymenial surface and narrower basidia measuring 30–55 × 4–6.5 µm (Zhu et al. 2024). *Tomentellaverruculata* X. Lu & H.S. Yuan is different from *T.velutina* by the arachnoid basidiomata, light brown to dark brown hymenial surface, and narrower basidiospores measuring 6.5–7.5 × 5.5–6 µm (Lu et al. 2022).

Phylogenetic analyses revealed that the new species *Tomentellawumenshanensis* was sister to *T.pallidobrunnea*. However, morphologically, *T.pallidobrunnea* is different from *T.wumenshanensis* by the pale brown to dark brown hymenial surface, thin-walled subhymenial generative hyphae and utriform, sinuous basidia (Yuan et al. 2020). Morphologically, the species *T.guiyangensis* H.S. Yuan & Y.Q. Zhu is distinct from *T.wumenshanensis* by its dark brown to chestnut hymenial surface and longer basidia measuring 35–55 × 5–9 µm (Zhu et al. 2024). *T.stipitobasidia* X. Lu & H.S. Yuan is distinguishable from *T.wumenshanensis* by arachnoid basidiomata with the brown to dark brown hymenial surface and longer basidia measuring 30–60 × 6–12 µm (Lu et al. 2022)

Phylogenetic analyses revealed that the new species *Tomentellayunnanensis* was grouped with three taxa, *T.bryophila*, *T.griseomarginata* and *T.subclavigera*. However, morphologically *T.bryophila* is distinguishable from *T.yunnanensis* by the yellow to ferruginous to reddish brown hymenial surface and yellowish to pale brown, nodose-septate generative hyphae (Reid and Larsen 1976). The species *T.griseomarginata* is distinct from *T.yunnanensis* by its greyish brown to dark brown hymenial surface, smaller basidia measuring 15–40 × 5–9 µm and smaller basidiospores measuring 6.5–7 × 6–6.5 µm (Yuan et al. 2020). The taxon *T.subclavigera* can be delimited from *T.yunnanensis* by having clavate cystidia measuring 105–145 × 7–11 µm and shorter basidia measuring 25–40 × 6–7.5 µm (Kõljalg 1996). Morphologically, *T.olivaceomarginata* H.S. Yuan & Y.Q. Zhu can be delimited from *T.yunnanensis* by having the pale brown to brown hymenial surface and shorter basidia measuring 15–35 × 6–8 µm (Zhu et al. 2024). *Tomentellacinereobrunnea* X. Lu & H.S. Yuan is distinguishable from *T.yunnanensis* by the greyish brown to brown hymenial surface and smaller basidia measuring 15–35 × 4–6 µm (Lu et al. 2022).

The phylogenetic tree reveals that individual species of *Tomentella* can form ectomycorrhiza with different host tree species in different families and closely related species in the same clade can be restricted to the same host tree family, in which the investigated forests were dominated by the coniferous trees *Pinuskesiya* mixed with families such as Ericaceae, Fagaceae, Lentibulariaceae, Orchidaceae and Rosaceae (Nguyen et al. 2012; Pócs et al. 2019). The present study found four new taxa in broad-leaved forests (Fagaceae and/or Pinaceae) mixed with coniferous trees. China is one of the most biodiverse countries in the world and more *Tomentella* species remain to be discovered here. Therefore, further studies are needed to enrich the species diversity of *Tomentella*.

## Supplementary Material

XML Treatment for Tomentella olivaceobasidiosa

XML Treatment for Tomentella velutina

XML Treatment for Tomentella wumenshanensis

XML Treatment for Tomentella yunnanensis

## ﻿Additional information

### Author ORCIDs

Xiaojie Zhang https://orcid.org/0000-0001-9954-3213

Fulei Shi https://orcid.org/0009-0003-8540-7079

Changlin Zhao https://orcid.org/0000-0002-8668-1075

### Data availability

All of the data that support the findings of this study are available in the main text.
